# Association of Atherosclerotic Peripheral Arterial Disease with Adiponectin Genes SNP+45 and SNP+276: A Case-Control Study

**DOI:** 10.1155/2013/501203

**Published:** 2013-06-03

**Authors:** Claudia D. Gherman, Doru Pamfil, Sorana D. Bolboacă

**Affiliations:** ^1^Department of Surgery, 2nd Surgical Clinic, “Iuliu Haţieganu” University of Medicine and Pharmacy Cluj-Napoca, 4-6 Clinicilor, 400006 Cluj-Napoca, Romania; ^2^Biotechnology Platform, University of Agricultural Sciences and Veterinary Medicine Cluj-Napoca, 3-5 Mănăştur, 400372 Cluj-Napoca, Romania; ^3^Department of Medical Informatics and Biostatistics, “Iuliu Haţieganu” University of Medicine and Pharmacy Cluj-Napoca, 6 Louis Pasteur, 400349 Cluj-Napoca, Romania

## Abstract

*Objectives*. We hypothesized that adiponectin gene SNP+45 (rs2241766) and SNP+276 (rs1501299) would be associated with atherosclerotic peripheral arterial disease (PAD). Furthermore, the association between circulating adiponectin levels, fetuin-A, and tumoral necrosis factor-alpha (TNF-*α*) in patients with atherosclerotic peripheral arterial disease was investigated. *Method*. Several blood parameters (such as adiponectin, fetuin-A, and TNF-*α*) were measured in 346 patients, 226 with atherosclerotic peripheral arterial disease (PAD) and 120 without symptomatic PAD (non-PAD). Two common SNPs of the ADIPOQ gene represented by +45T/G 2 and +276G/T were also investigated. *Results*. Adiponectin concentrations showed lower circulating levels in the PAD patients compared to non-PAD patients (*P* < 0.001). Decreasing adiponectin concentration was associated with increasing serum levels of fetuin-A in the PAD patients. None of the investigated adiponectin SNPs proved to be associated with the subjects' susceptibility to PAD (*P* > 0.05). *Conclusion*. The results of our study demonstrated that neither adiponectin SNP+45 nor SNP+276 is associated with the risk of PAD.

## 1. Introduction

Peripheral arterial disease (PAD) is a chronic arterial disease of atherosclerosis of the extremities causing ischemia [1]. Smoking, high blood pressure, atherosclerosis, and high cholesterol as well as the age over 60 years old are the main known risk factors of PAD [1]. The PAD affects equally men and women [2], and its prevalence increases with age [3]. The classic symptom of intermittent claudication occurs only in ~11% [4]; 50% complained of a variety of leg symptoms [5], and ~40% does not complain of any leg pain [3]. The prevalence of PAD had different values, from 4.4% in people ≥40 years of age in Canada [6], 7% in Belgium population, 8.1% in Dutch general population, 12.2% in France, and 22.9% in Italy to 28% in Greece [7], while in Romania it is 18.7% [8]. PAD could be seen as a marker for systemic atherosclerotic disease, leading to fatal (such as death) or nonfatal events (such as cardiovascular events) [2, 9].

Adiponectin is an adipocytokine with anti-inflammatory and antiatherogenic effects, secreted especially by the adipose tissue, which is considered to be an active endocrine organ [10, 11]. By inhibiting the tumoral necrosis factor-alpha (TNF-*α*), it reduces the expression of the cellular adhesion molecules in the endothelium and has a series of other antiatherogenic effects [12]. Adiponectin accumulates in the wall of the injured arteries, suppresses the accumulation of lipids, and inhibits the transformation of the macrophages into foamy cells, as well as the proliferation of the smooth muscle cells, a succession of phenomena encountered in the initiation and evolution of atherosclerotic lesions [13].

The plasma levels of adiponectin are low in obesity, type 2 diabetes mellitus (DM), insulin resistance, dyslipidemia, coronary artery disease (CAD), and peripheral arterial disease (PAD) [14–16]. Fetuin-A (former name for the human protein: *α*_2_-Heremans-Schmid glycoprotein (AHSG)) is an abundant serum protein that is exclusively produced by the liver, tongue, and placenta [17]. Besides these well-documented effects of fetuin-A on the insulin receptor of muscle and liver, this protein may induce whole-body insulin resistance as an action on adipose tissue [17]. Moreover, human plasma fetuin-A levels are associated with the metabolic syndrome and an atherogenic lipid profile [17, 18]. These states are characterized by subclinical inflammation and hypoadiponectinemia [17, 19], so fetuin-A might influence adiponectin production.

Circulating levels of adiponectin are also determined by genetic factors. The adiponectin gene ADIPOQ is considered the major gene influencing adiponectin concentration with single nucleotide polymorphisms (SNPs) in its coding region and promoter [20]. Human adiponectin is encoded by the ADIPOQ gene on the chromosomal locus 3q27, next to gene encoding human fetuin-A [21]. It shares structural similarities with the complement C1q protein and the TNF family, both having an important role in the inflammation, immune system, and atherosclerosis [22]. In the genome-wide scans, more than 10 SNPs were reported. Of these, polymorphisms +45 in exon 2 and +276 in intron 2 were frequently associated with DM, obesity, and CAD [11, 23, 24].

Considering that PAD could have mutilating consequences (such as major amputations) or even more could be potentially fatal, we aimed to relate the soundly studied SNP+45 (rs2241766) and SNP+276 (rs1501299) adiponectin polymorphisms in relation to PAD in a sample of Romanians. Additionally, we also evaluated the association of adiponectin levels with fetuin-A and, respectively, TNF-*α* in patients with atherosclerotic peripheral arterial disease.

## 2. Materials and Methods

### 2.1. Study Design and Participants

An observational case-control study was conducted between December 15, 2009 and October 15, 2011 among adult patients treated in the 2nd Surgery Clinic of the County Clinical Hospital in Cluj-Napoca, Romania.

There were included in the PAD group all patients that complained of leg symptoms with exertion or ischemic rest pain, and/or tissue loss, and who were diagnosed with atherosclerotic PAD using Doppler examination and ankle brachial pressure index (ABI, the ratio of the blood pressure in the lower legs to the blood pressure in the arms) (ABI ≤ 0.9) [25]. For almost two patients who accomplished the inclusion criteria in the PAD group and agreed with participation in our study, one non-PAD patient was included in the non-PAD group. Controls (non-PAD patients) were age- and gender-matched outpatients who referred to the same hospital for lower limb chronic venous insufficiency or general surgery problems, with ABI > 0.9.

Both PAD and non-PAD patients were excluded if they had known serious and/or chronic illnesses or demonstrated clinical, biochemical, or hematological proof of cardiovascular, hepatic, or renal failure.

All patients included in our study signed an informed consent for participation. The study was approved by the Local Ethics Committees, and it was in accordance with the Helsinki Declaration.

### 2.2. Anthropometric and Biochemical Analyses

Anthropometric parameters represented by weight and height were measured for each patient included in the study. The body mass index was calculated by applying the following formula: BMI = weight (kg)/height (m^2^) [26]. Data about the medical history of each patient regarding the presence of arterial hypertension (AHT), obesity, diabetes mellitus (DM), dyslipidemia, and coronary artery disease (CAD) were also collected. Beside the medical history, all patients were asked at the time of inclusion in the study if they were or not active smokers.

The following biochemical parameters as predictors were determined from the blood sample for each subject included in the study: cholesterol, triglycerides (TG, mg/dL), fibrinogen (mg/dL), high density lipoprotein (HDL, mg/dL), glycemia (mg/dL), creatinine (mg/dL), and C-reactive protein (CRP, mg/dL—using standard enzymatic method). The assay was performed using a COBAS MIRA Plus analyzer Hoffmann-La Roche (Diagnostic reagents, Budapest, Hungary) and Sysmex CA-1500 System. Circulating plasma levels of adiponectin, fetuin-A, and TNF-*α* were measured by a commercially available method, using Quantikine reagents (R&D Systems, Minneapolis, USA).

### 2.3. Adiponectin SNP+45 and SNP+276 Polymorphisms Determination

**Figure 1:**
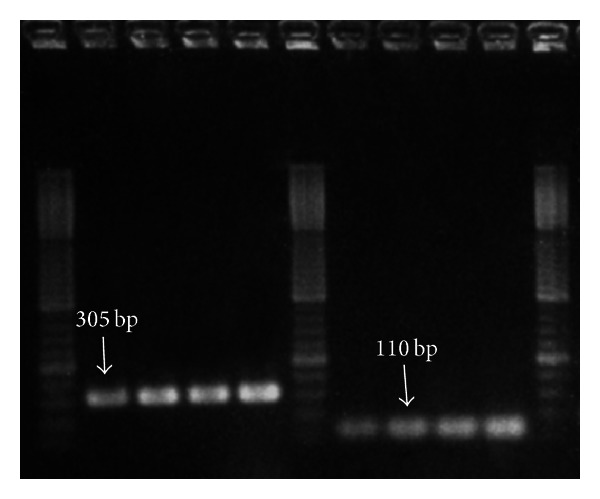
Electrophoretic profile of amplification products obtained with primers used for further highlight of SNPs+45T/G and SNP+276G/T by enzymatic digestion.

**Figure 2:**
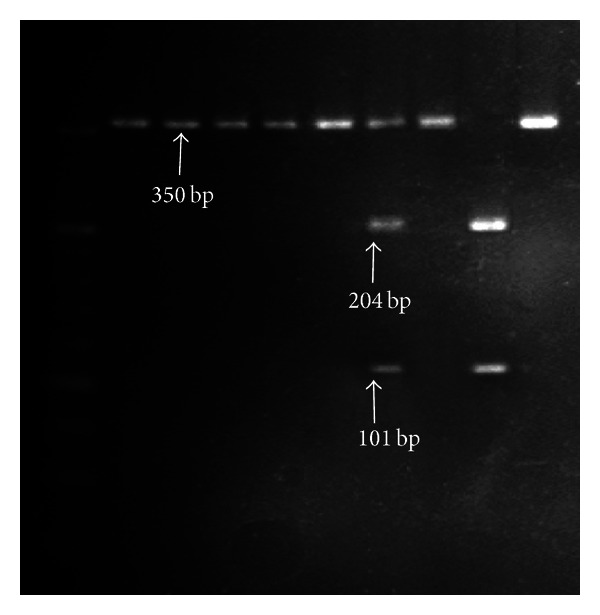
Fast digest restriction endonucleases with Ava I for SNP+45 T/G.

**Figure 3:**
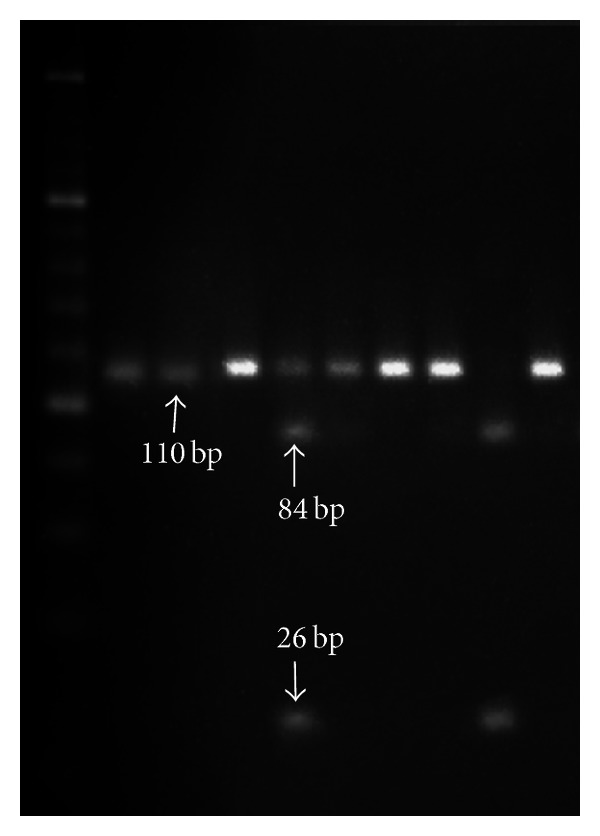
Fast digest restriction endonucleases with Hinf I for SNP+276 G/T.

Peripheral blood (5 mL) for DNA extraction was collected in tubes containing EDTA, both for PAD and non-PAD groups. DNA was isolated using a MagNA Pure LC DNA Isolation Kit I (Roche) on the MagNA Pure LC platform (Roche), applying the producer's protocol. DNA concentration and purity were assessed using the NanoDrop ND1000 (Thermo Scientific). A polymerase chain reaction-restriction fragment length polymorphism (PCR-RFLP) assay, using primers previously published in [27], was employed to genotype the 45T/G and 276G/T polymorphisms. The amplification was performed in a volume of 20 *μ*L, with 100 ng DNA, 1X PCR buffer, 2.5 mM MgCl_2_, 0.2 mM of each dNTP, 0.5 uM of each primer, and 0.5 U Taq DNA polymerase (Promega), on a Corbett Research Palm-Cycler. The following steps were applied for amplification: initial denaturation at 94°C for 5 min, 35 cycles of 94°C for 30 s, 62°C for 30 s (SNP+45 T/G), respectively, 55°C for 30 s (SNP+276 G/T), 72°C for 50 s, and a final elongation step at 72°C for 10 min. 5 *μ*L of the PCR products was digested with 0.5 FDU of Fast Digest restriction endonucleases (Fermentas) (Ava I for SNP+45 T/G and Hinf I for SNP+276 G/T, resp.) for 5 min at 37°C. The PCR products of digestion were analyzed by electrophoresis on 2% and 4%, respectively, agarose gels stained with ethidium bromide. The fragments obtained for SNP+45 T/G had 305 bp for the GG genotype, 204 and 101 bp for the G/T genotype (Figure 1) and 305, 204, and 101 bp for the G/T genotype (Figure 2). The fragments obtained for SNP+276 G/T were 110 bp for the GG genotype, 84 and 26 bp for the G/T genotype, and 110, 84, and 26 bp for the G/T genotype (Figure 3).

### 2.4. Statistical Analysis

Qualitative variables were summarized as percentages and associated with 95% confidence intervals (95% CI, provided in squared brackets) calculated with an optimized binomial formula similar to that presented in [28]. The main characteristics of the metric variables were expressed as mean and standard deviation (*m* ± s.tdev.) whenever data were normally distributed; otherwise, median and interquartile ranges (median (*Q*1; *Q*3), where *Q*1 = 25th percentile; *Q*3 = 75th percentile) were used. Student's *t*-test was applied to compare continuous variables of two groups whenever data proved to be normally distributed; otherwise, Mann-Whitney test was applied. Significant differences in continuous variables among more than two groups were confirmed by the ANOVA test whenever data proved to be normally distributed; otherwise, the Mann-Whitney *U* test was applied.

First step in genetic analysis was represented by the verification of the Hardy-Weinberg equilibrium by applying the chi-squared goodness-of-fit test performed using DeFinetti program (http://ihg.gsf.de/cgi-bin/hw/hwa1.pl). Adjusted odds ratio (OR) according to age and gender and their 95% confidence intervals (95% CI) were estimated using logistic regression technique. Furthermore, logistic regression analysis was also applied to assess the effect of the SNP+45 and SNP+276 polymorphisms on PAD after adjustment for covariates (represented by those variables that proved to be significantly different in PAD group compared to non-PAD group) taking into consideration those metric variables that proved to be statistically different between the investigated groups.

Statistical analysis was performed using the SPSS software version 16 (SPSS Inc., Chicago, IL, USA). All *P* values were two tailed and were considered as significant when lower than 0.05. When more than two means were compared, the *P*-value was adjusted according to the number of investigated subgroups, and thus values smaller than 0.0167 were considered statistically significant.

## 3. Results

Three hundred forty-six patients were included in our study, two hundred and twenty-six in PAD group and one hundred and twenty in non-PAD group. Baseline characteristics of patients by groups are summarized in Table 1. The subjects with PAD proved to have higher values of serum triglycerides (*P* = 0.028, see Table 1), while serum fibrinogen values proved to be lower in PAD group compared to non-PAD group (*P* < 0.001, see Table 1) but with normal values in both groups.

Hypoadiponectinemia has been observed in PAD group (5.66 ± 1.37) compared to controls (6.55 ± 1.23) (*t*-test: statistics = −6.165, *P*-value < 0.001). Furthermore, high fetuin-A levels have been observed in PAD group (463.60 ± 124.42) compared to controls (368.31 ± 87.83) (*t*-test: statistics = 7.459, *P*-value < 0.001). As far as TNF-*α* was concerned, statistically significant difference between groups was identified (PAD group = 1.14 ± 0.45; non-PAD group = 1.04 ± 0.45; *t*-test: statistics = 2.105, *P*-value = 0.0360), but this difference has no clinical significance since the values in both groups are within normal range (0.550–2.816 pg/mL).

Regarding the SNP+45 and SNP+276 polymorphisms, deviation from Hardy-Weinberg equilibrium was not identified neither for SNP+45 (*F*_non-PAD_ = 0.14802, *P*-value_non-PAD_ = 0.1056; *F*_PAD_ = 0.09660, *P*-value_PAD_ = 0.1369) nor for SNP+276 (*F*_non-PAD_ = 0.14869, *P*-value_non-PAD_ = 0.1271; *F*_PAD_ = 0.0387, *P*-value_PAD_ = 0.5785). The genotypes frequencies according to the groups are presented in Table 2.

The results obtained on testing the susceptibility to PAD inheritance caused by adiponectin SNP+45 and SNP+276 polymorphisms are presented in Table 3. 

The susceptibility to PAD inheritance caused by adiponectin SNP+45 and SNP+276 polymorphisms was tested using logistic regression analysis. The forward LR method was applied to exclude the effects of confounders (such as gender, age, smoking status, DM, obesity, AHT, CAD, antihypertensive therapy, and biochemical parameters like cholesterol, HDL, TG, glycemia, fibrinogen, adiponectin, fetuin-A, TNF-*α*, duration of diabetes, oral hypoglycemic therapy, insulin therapy, oral and insulin therapy, and antihypertensive and statins therapy) as determinants other than adiponectin SNP+45 and SNP+276. The forward Wald method revealed that the intercept was not significant in any model, and as a result, the reported model is a model without intercept. The obtained results showed that the analyzed adiponectin SNP+45 and SNP+276 are not associated with PAD (Table 4).

A test of the full model against a constant model was statistically significant (chi-squared statistics = 149, *P* = 7.9 · 10^−29^, df = 7). The model presented in Table 4 indicates that almost 47% of the variation in PAD is explained by the logistic model (Nagelkerke *R* ^2^ = 0.467), the model being able to correctly classify patients as PAD and non-PAD in 75.4% of the cases (85% for PAD patients and 57.5% of non-PAD patients).

The association of adiponectin, fetuin-A, and TNF-*α* with status of PAD or non-PAD was also investigated, and the obtained results according to the studied gene polymorphisms are presented in Table 5 for SNP+45 and in Table 6 for SNP+276.

## 4. Discussion

The association of plasma adiponectin levels with two adiponectin SNPs and plasma fetuin-A and TNF-*α* levels was successfully examined in a group of patients with PAD, using a group of controls.

The patients included in investigated groups were similar in the majority of investigated characteristics with some exceptions (see Table 1). The prevalence of the investigated disease of our studied sample proved to be in lines with the specialty literature, males and elderly persons being most frequently affected by PAD [29]. As far as associated diseases were concerned, a significantly higher proportion of patients were with arterial hypertension (*P* = 0.024) and coronary artery disease (*P* = 0.005) in PAD group compared to non-PAD group. Note that atherosclerosis is at the basis of arterial hypertension, coronary artery disease, and peripheral arterial disease and the link between arterial hypertension and peripheral arterial disease, has already been identified [29]. Neither the proportion of obese subject nor the BMI was significantly different between PAD and non-PAD groups (Table 1, *P*-values > 0.6). Furthermore, no significant differences were identified between investigated groups in terms of diabetes duration and treatment or antihypertensive agents and statins (Table 1).

Triglycerides proved to be significantly higher in PAD group compared with non-PAD group (*P* = 0.028, Table 1), while fibrinogen level proved to be significantly lower in PAD group compared with non-PAD group (*P* < 0.001, Table 1). Note that even if the values of triglycerides and fibrinogen were significantly different, these differences have no clinical significance since the values are within normal ranges in both groups. 

The relation between smoking and peripheral arterial disease had already been identified [28], so it was not a surprise to identify a significantly higher proportion of smokers within PAD group compared to non-PAD group (*P* < 0.001). 

The lower values of adiponectin in PAD group compared to non-PAD group (*P* < 0.001) identified in our study are in agreement with the results obtained by other researchers [16, 30]. It is well known that some drugs and some antidiabetic drugs such as thiazolidinedione [31] as well as insulin [32] had influences on serum adiponectin levels. However, the s with diabetes included in our study were treated with biguanides or sulfonylurea drugs, or insulin, or a combination of oral and insulin therapyies and thus these therapeutic strategies could have an effect on the serum adiponectin levels. But since no significant differences of proportion of subjects with diabetes or the proportion of subjects that followed different antidiabetic therapeutic schemas were identified, the identified hypoadiponectinemia in the PAD group compared to non-PAD group could not be attributed to antidiabetic treatment. 

The results of our study showed significantly higher levels of both fetuin-A in PAD group compared to controls (*P* < 0.001), hyperfetuinemia-A being previously identified in patients with type 2 diabetes and peripheral arterial disease [33]. 

No genotype differences in terms of SNP+45 or SNP+276 were identified between PAD and non-PAD groups (Table 2). It could be noted that none of these two investigated variants emerged from recent genome-wide association studies [34, 35].

The link between susceptibility to PAD inheritance caused by adiponectin SNP+45 and SNP+276 polymorphisms was investigated in our study, but we did not identify any risk allele neither for SNP+45 nor for SNP+276. According to our results (Table 3), the odds ratio ranged between 0.667 (SNP+276) and 1.622 (SNP+45), and all associated 95% confidence intervals comprised the value of 1; thus, the results are not suitable for generalization. Since none of these results was statistically significant, it could be concluded that adiponectin SNP+45 and SNP+276 polymorphisms are not a risk factor for PAD even if previous studies identified the link between these polymorphisms and cardiovascular conditions [36, 37]. 

Logistic regression analysis was used to exclude the effect of other determinants of PAD different by adiponectin SNP+45 and SNP+276 genotypes. Four biochemical parameters represented by glycemia, fibrinogen, fetuin-A, and TNF-*α* as well as smoking, CAD, and antihypertensive therapy proved significantly linked with PAD (see Table 4). But it could be noted that just a small part of the variation in PAD could be explained by the identified logistic model (Nagelkerke *R* ^2^ = 0.467). The identified model did not include the values of adiponectin; however, inconsistent results were reported in terms of the impact of SNP+45 on the blood level of adiponectin [38].

The association of adiponectin, fetuin-A, and TNF-*α* with status of PAD or non-PAD was furthermore investigated in relation to the SNPs genotypes. Just one significant result (*P* < 0.0167) was obtained in this analysis, and this result refers only to the non-PAD group: adiponectin had significantly higher values in SNP+45 GG homozygote compared to SNP+45 TG heterozygote (Table 5, *P* = 0.015).

The investigation of the association to atherosclerotic peripheral arterial disease with adiponectin genes SNP+45 and SNP+276 was conducted as an observational study that is one of the limitations of our study. Other main limitations of our study must be mentioned. First limitation is given by the small investigated samples and the control : case ratio that led to very small power of this analysis that varied from 5.0784 for SNP+45-G as risk allele to 27.7967 for SNP+45-T as risk allele. Second limitation is given by the lack of replication due to the absence of the investigated SNPs from the recent genome-wide association studies of type 2 diabetes [34, 35], replication that is fundamental for genetic studies. However, well-designed observational studies could be valuable researches, and here probably a matched case-control study could provide results not affected by bias introduced by absence of matches in terms of AHT and CAD, which both are related to atherosclerosis that is the main cause of PAD. Some factors such as ABI threshold, which could bring a potential in missing patients with mild PAD, can induce a classification bias within investigated sample. Furthermore, an analysis taking into consideration the ethnicity could bring useful information in this field since adiponectin genetic variants may represent heterogeneity among populations [39]. Finally, confirmation of our findings in larger samples considering also the heterogeneity across different ethnicities is welcomed.

As far as we know, the assessment of atherosclerotic peripheral arterial disease with adiponectin gene SNP+45 and SNP+276 was not investigated until now. Thus, this is the first study that was conducted to investigate if any link exists between adiponectin gene SNP+45 and SNP+276 and atherosclerotic peripheral arterial disease. The continuation of the study on ever larger groups of patients and/or on matched samples as well as the assessment of additional genetic parameters could fetch new, more relevant data or could confirm the data obtained so far.

## 5. Conclusions

The main findings of the study can be summarized as follows: (i) low levels of adiponectin were observed in patients with PAD; (ii) high levels of fetuin-A were observed in patients with PAD; (iii) neither SNP+45 nor SNP+276 proved to be associated with PAD; (iv) PAD proved to be significantly related to glycemia, fibrinogen, fetuin-A, TNF-*α*, smoking, coronary artery disease and antihypertensive therapy in a logistic regression model with an accuracy of 75.7%.

## Figures and Tables

**Table 1:** Anthropometric and biochemical characteristics by groups.

Variable	PAD group (*n* = 226)	Non-PAD group (*n* = 120)	Comparison between groups *P*-value
Gender: male^∗,a^	82 [77, 87]	80 [72, 87]	0.603
Smokers^∗,a^	62 [56, 68]	43 [33, 52]	<0.001
DM^∗,a^	33 [27, 39]	32 [23, 41]	0.840
Obesity^∗,a^	20 [15, 26]	22 [15, 30]	0.665
AHT^∗,a^	61 [54, 67]	48 [39, 57]	0.024
Antihypertensive agents^a^	53 [46, 60]	42 [33, 51]	0.049
CAD^∗,a^	53 [46, 60]	37 [28, 46]	0.005
Age (years)^∗∗,b^	63 (56; 74)	62 (57; 71)	0.626
BMI (kg/m^2^)^∗∗,b^	25.82 (23.12; 29.07)	25.73 (22.71; 29.34)	0.896
Cholesterol (mg/dL)^∗∗,b^	194 (167; 230)	191 (157; 223)	0.139
TG (mg/dL)^∗∗,b^	113 (89; 140)	104 (80; 139)	0.028
HDL (mg/dL)^∗∗,b^	49 (42; 56)	48 (41; 55)	0.356
Statins^a^	75 [69, 81]	72 [63, 79]	0.549
Glycemia (mg/dL)^∗∗,b^	96 (86; 126)	96 (86; 108)	0.207
Diabetes duration^a^			
≤5 years	7 [4, 11]	8 [3, 13]	0.739
5–10 years	13 [9, 18]	13 [8, 21]	>0.999
>10 years	12 [8, 17]	9 [4, 16]	0.376
Diabetes treatment^a^			
Oral hypoglycemic agent***	8 [4, 12]	10 [5, 17]	0.542
Insulin therapy	17 [12, 22]	17 [10, 24]	>0.999
Oral and Insulin therapy	7 [4, 11]	4 [2, 9]	0.224
Creatinine (mg/dL)^∗∗,b^	0.90 (0.70; 1.00)	0.90 (0.70; 1.00)	0.094
Fibrinogen (mg/dL)^∗∗,b^	306 (249; 367)	343 (310; 387)	<0.001^c^
CRP (mg/dL)^∗∗,b^	0.70 (0.50; 0.90)	0.90 (0.60; 1.00)	0.816

*% [95% CI]; **median (Q1; Q3), where Q1 = 25th percentile; Q3 = 75th percentile; ***biguanides or sulfonylurea; ^a^ *P* value associated with *Z*-test; ^b^ *P*-value associated with Mann-Whitney test; ^c^normal limits for both groups; DM: diabetes mellitus; AHT: arterial hypertension; CAD: coronary artery disease; BMI: body mass index; TG: triglycerides; HDL: high density lipoprotein; CRP: C-reactive protein.

**Table 2:** Genotypic frequencies of adiponectin SNP+45 and SNP+276 polymorphism in Romanians PAD and non-PAD patients.

Group	Genotype—% [95% CI]		
	GG	TT	TG
SNP+45			
PAD*	3 [1–6]	77 [71–83]	20 [15–26]
Non-PAD	3 [1–8]	77 [68–84]	19 [13–27]
*Z* (*P*-value)	−0.33 (0.7388)	0.16 (0.8731)	0.17 (0.8685)
SNP+276 *n* (%)			
PAD*	59 [53–66]	35 [28–41]	6 [4–10]
Non-PAD	61 [52–70]	31 [23–40]	8 [4–15]
*Z* (*P*-value)	−0.28 (0.781)	0.70 (0.485)	−0.72 (0.474)

%: percentage; [95% CI]: 95% confidence interval; *Z*: *Z*-test for comparison of two proportions.

**Table 3:** Association analysis for adiponectin SNP+45 and SNP+276 polymorphisms and PAD adjusted by age and gender.

Statistics	Risk allele	Genotype	Genotype
SNP+45			

	T	TT	TG

OR	1.276	1.637	1.622
[95% CI]	[0.352–4.621]	[0.484–5.529]	[0.444–5.925]
*P*-value	n.s.	n.s.	n.s.

	G	GG	TG

OR	0.998	n.a.	n.a.
[95% CI]	[0.587–1.698]		
*P*-value	n.s.		

SNP+276			

	G	TT	GT

OR	1.387	0.885	0.667
[95% CI]	[0.595–3.231]	[0.543–1.442]	[0.271–1.644]
*P*-value	n.s.	n.s.	n.s.

	T	GG	GT

OR	1.050	1.327	1.499
[95% CI]	[0.665–1.658]	[0.560–3.147]	[0.608–3.695]
*P*-value	n.s.	n.s.	n.s.

OR: odds ratio; [95% CI]: 95% confidence interval; *P*-value: significance associated with OR; n.s.: not statistically significant; n.a.: insufficient data to carry out the analysis.

**Table 4:** Logistic regression model: results.

Parameter	*B*	S.E.	W(*p*)	Exp(*B*)
Glycemia	−0.011	0.004	6.255 (1.24 · 10^−2^)	0.989 [0.981–0.998]
Fibrinogen	0.008	0.002	26.12 (3.21 · 10^−7^)	1.008 [1.005–1.011]
Fetuin-A	−0.012	0.002	49.32 (2.18 · 10^−12^)	0.988 [0.985–0.992]
TNF-*α*	1.407	0.402	12.27 (4.61 · 10^−4^)	4.084 [1.858–8.973]
Smoking	1.004	0.280	12.83 (3.41 · 10^−4^)	2.730 [1.576–4.729]
CAD	0.958	0.279	11.805 (5.91 · 10^−4^)	2.606 [1.509–4.501]
Antihypertensive therapy	0.577	0.268	4.657 (3.09 · 10^−2^)	1.781 [1.054–3.010]

*B*: logistic coefficients at degree of freedom (df) = 1; SE: standard error; W(*p*): Wald statistics and associated probability; CAD: coronary artery disease.

**Table 5:** Comparisons on adiponectin, fetuin-A, and TNF-*α* by groups according to SNP+45.

Group	Variable	SNP+45			ANOVA Test	
		GG (*n* = 6)	TT (*n* = 175)	TG (*n* = 45)	Stat	*P*-value
PAD	Adiponectin (pg/mL)	6.17	5.74	5.29	2.352	0.100
	Fetuin-A (*µ*g/mL)	466.33	455.05	496.49	2.004	0.137
	TNF-*α* (pg/mL)	1.17	1.11	1.25	1.626	0.199

		GG (*n* = 4)	TT (*n* = 93)	TG (*n* = 23)		

Non-PAD	Adiponectin (pg/mL)	7.88^a^	6.62	6.46^a^	4.819	0.010
	Fetuin-A (*µ*g/mL)	344.25	366.45	380.00	0.371	0.691
	TNF-*α* (pg/mL)	0.89	1.03	1.09	0.374	0.689

PAD: peripheral arterial disease; Stat: statistical parameter of ANOVA test.

^a^Bonferroni test, mean difference = 1.84630, *P* = 0.015 (adjusted *P*-value considered statistically significant < 0.0167).

**Table 6:** Comparisons on adiponectin, fetuin-A, and TNF-*α* by groups according to SNP+276.

Group	Variable	SNP+276			ANOVA test	
		GG (*n* = 134)	TT (*n* = 14)	GT (*n* = 78)	Stat	*P*-value
PAD	Adiponectin (pg/mL)	5.75	5.67	5.51	0.735	0.480
	Fetuin-A (*µ*g/mL)	452.34	455.86	484.33	1.668	0.191
	TNF-*α* (pg/mL)	1.08	1.24	1.23	3.094	0.047

		GG (*n* = 73)	TT (*n* = 10)	GT (*n* = 37)		

Non-PAD	Adiponectin (pg/mL)	6.74	6.33	6.49	0.279	0.757
	Fetuin-A (*µ*g/mL)	362.68	368.30	379.41	0.441	0.645
	TNF-*α* (pg/mL)	1.04	0.90	1.07	0.608	0.546

PAD: peripheral arterial disease; Stat: statistical parameter of ANOVA test.

## Abbreviations

PAD: Peripheral arterial disease
TNF-*α*: Tumoral necrosis factor-alpha
SNPs: Single-nucleotide polymorphisms
DM: Diabetes mellitus
CAD: Coronary artery disease
ABI: Ankle-brachial index
BMI: Body mass index
AHT: Arterial hypertension
TG: Triglycerides
HDL: High density lipoprotein
CRP: C-reactive protein
ELISA: Enzyme-linked immunosorbent assay
PCR-RFLP: Polymerase chain reaction-restriction fragment length polymorphism
95% CI: Confidence intervals at a significance level of 5%.
